# Implications of systemic racism in emergency medical services: On prehospital bias and complicity

**DOI:** 10.1016/j.eclinm.2022.101525

**Published:** 2022-06-25

**Authors:** Christian Angelo I. Ventura, Edward E. Denton, Benjamin R. Asack

**Affiliations:** a Department of Graduate Medical Sciences, Boston University School of Medicine, Boston, MA USA; Department of Allied Health, Roxbury Community College, Roxbury Crossing, MA, USA; b Department of Emergency Medicine, University of Arkansas for Medical Sciences, Little Rock, AK, USA; c University of Vermont, Burlington, VT, USA

Public health research has increasingly proven racial disparities within emergency medicine, as exemplified by the fallibility of pulse oximeters resulting in unreliable detection of respiratory compromise, to implicitly biased clinical perception of pain in Black and Brown patients.^1^ There is a lack of literature detailing the complicity of Emergency Medical Services (EMS) clinicians who sit among a nation with an inequitable healthcare system. In this piece, we aim to shed light on how disparities in prehospital emergency medical care harm patients of color. Informed by Black, Indigenous, and People of Color (BIPOC) accounts, our experiences as prehospital clinicians, and available literature, we discuss pressing concerns with actionable implications. We argue that patients of color, and particularly Black and Brown patients, are disproportionately subject to faulty clinical assessment and treatment by EMS largely due to provider implicit bias and institutionally-embedded racism inherent within the American healthcare system.

EMS clinicians are trained to conduct a holistic assessment encompassing subjective complaints, quantitative values, physiological indicators, and intuitive-based discretion. But how do we account for the realization that we often fail to teach and remind clinicians that cyanosis, a late indicator of respiratory failure, does not typically present as blue-tinted skin in Black patients, but rather gray or white? How do we advise clinicians to employ pulse oximetry as a reliable tool for assessing a patient's respiratory status, when we know oximetry readings are more likely to be inaccurate in Black hypoxic patients^1^? These concerns have become increasingly urgent due to the rising opioid epidemic that disproportionately affects Black and Brown communities, which the COVID-19 pandemic has only exacerbated.^2^

A 2014 study in a journal published by the American Psychological Association suggests that Black boys are often perceived as less innocent and older than their White counterparts.^3^ Similar phenomena have been well-studied among medical professionals, where they have reported inaccurate beliefs regarding biological differences between Black and White patients, as well as significant disparities in pain assessment and treatment of Black patients rooted in racial bias.^4^ In this same study, medical students and residents had endorsed beliefs such as “Blacks’ nerve endings are less sensitive than whites,’” “Black people's blood coagulates more quickly than whites',” and “Blacks’ skin is thicker than whites’” A 2019 study of EMS agencies in Oregon found that racial minorities were less likely to receive pain assessments and pain medication in the 911 setting.^5^

The death of 23-year-old Elijah McClain on August 30, 2019 sparked global discussion of EMS involvement in police-related deaths of Black individuals. When a stop-and-frisk of a Black man escalated to a stranglehold, EMS was requested on scene and paramedics subsequently administered ketamine, an intervention approved by Aurora EMS protocols to treat excited delirium.^6^ While much has been made of the role of ketamine, has EMS adequately discussed the role of racial bias at the initial steps of patient assessment? Prior to injection, McClain received no clinical assessment, was already physically restrained by police, sustained a carotid chokehold and briefly became unconscious. Immediately on scene, EMS is trained to assess the ABCs – airway, breathing, and circulation. In police footage, McClain is heard repeatedly saying he cannot breathe, a critical observation for clinicians if administering ketamine. Did racial bias impede their review of the five rights of drug administration? Further, the medical community has continued to question the validity of excited delirium as a legitimate medical diagnosis.^7^

While systemic racism is not unique to EMS, it is worth investigating its industry-specific impact. As we continue to build knowledge of disparities in prehospital care, there are actions we can take that are proven effective. At the systemic level, we invoke National EMS Education Standards to mandate adequate training in implicit bias, clinical assessment and treatment variances for patients of color, and for state and local EMS authorities to follow suit. We believe that this competency should also be assessed by the national registry EMT exam. In addition, we call for radical efforts to diversify the profession by campaigns to train and employ racial minorities, as approximately 70% of EMTs and paramedics are White.^8^ While we acknowledge that racial injustice in EMS manifests itself beyond solely clinical care, these critical actions have tremendous potential to move the industry forward towards evidence-based care for everyone.

## Contributors

All authors commented critically in the manuscript, revisions were made, and the final draft was prepared and submitted.

## Funding

The work is not funded by any specific source.

## Declaration of interests

The investigators report no known conflicts of interest, financial or otherwise.

The work is solely that of the authors and does not necessarily represent the views, policies, or opinions of their affiliated institutions, employers, or partners. It was not reviewed or endorsed by any specific institution in particular.
